# Transnational and Local Co-ethnic Social Ties as Coping Mechanisms Against Perceived Discrimination - A Study on the Life Satisfaction of Turkish and Moroccan Minorities in the Netherlands

**DOI:** 10.3389/fsoc.2021.671897

**Published:** 2021-06-28

**Authors:** Ece Arat, Özge Bilgili

**Affiliations:** ^1^ Department of Sociology, Faculty of Social and Behavioural Sciences, Utrecht University, Utrecht, Netherlands; ^2^ European Research Centre on Migration and Ethnic Relations (ERCOMER), Faculty of Social and Behavioural Sciences, Utrecht University, Utrecht, Netherlands

**Keywords:** perceived discrimination, coping mechanisms, transnational co-ethnic social ties, local co-ethnic social ties, the Netherlands, life satisfaction

## Abstract

Perceived ethnic discrimination is known to decrease minorities’ life satisfaction. This research investigates the extent to which minorities’ local and transnational co-ethnic social ties mitigate the negative effects of perceived discrimination on life satisfaction. Put differently, focusing on the experiences of Turkish and Moroccan minorities, we discuss whether co-ethnic social ties, both locally and transnationally embedded, can be considered as coping mechanisms against perceived discrimination. Furthermore, we investigate whether these mechanisms work differently for first- and second-generation minorities. Using Netherlands Longitudinal Life-course Study, we reveal that perceived discrimination is positively associated with local co-ethnic social ties in Netherlands which consequently predicts higher life satisfaction for both generations. Surprisingly, we also show that only among the second generation perceived discrimination is associated with stronger transnational co-ethnic social ties, but not the first generation. Having these transnational ties however are beneficial for life satisfaction of both generations. Consequently, we highlight the importance of recognizing transnational embeddedness of minorities and studying the effects transnational co-ethnic social ties on subjective well-being outcomes especially for second-generation minorities.

## Introduction

Ethnic minority groups face discrimination in many European countries (Eurobarometer, 2019). As reported by the Netherlands Institute for Social Research, ethnic origin is an important determinant of perceived discrimination in the Netherlands (Andriessen et al., 2020). Perceived discrimination refers to feelings of adverse and unequal treatment in intergroup interactions due to characteristics such as age, gender, and ethnicity (Alanya et al., 2017). Perceiving discrimination based on ethnic origin negatively affects minorities’ psychological well-being in general (Schmitt et al., 2014; Sirin et al., 2015) and their life satisfaction in particular (Verkuyten, 2008). Research has shown that ethnic minorities have lower levels of life satisfaction than native groups (Arpino and De Valk, 2018)—a difference that also remains for the second generation (Safi, 2010; De Vroome and Hooghe, 2014). This is problematic as, in addition to being a well-being outcome (Outten and Schmitt, 2015), life satisfaction also associates with higher income, better interpersonal relationships, and health conditions (Lyubomirsky et al., 2005).

There are different coping mechanisms helping individuals deal with the negative impacts of discrimination on their well-being (Brittian et al., 2013; Kuo, 2014), such as increased identification with one’s in-group (Branscombe et al., 1999), positive reappraisal, or seeking social support (Tandon et al., 2013). As suggested by Cohen (2004), social support increases the ability to deal with stressful experiences—meaning that, in addition to being a coping strategy in itself, social support can enable or strengthen other types of coping strategies. This article, therefore, focuses on social support as a coping mechanism to mitigate the harmful effects of perceived discrimination on minorities’ life satisfaction. Namely, we investigate how transnational co-ethnic social ties with origin country and local co-ethnic social ties in the destination country suppress the negative effects of perceived discrimination on life satisfaction among both first- and second-generation Turkish and Moroccan minorities in the Netherlands. We define co-ethnic social ties as contacts between people belonging to the same ethnic group.

Prior research focusing on ethnic minorities' social ties has often examined the ties with the host society—taking them as an indicator of integration into the destination country (e.g. Vervoort and Dagevos, 2011). The much smaller number of studies referring to co-ethnic ties is usually limited to local ones located in the destination country. Research on local co-ethnic social ties in the destination country assumes that geographical location is a critical determinant to provide more direct social support (McPherson et al., 2001; Jasinskaja-Lahti et al., 2006).These studies often study how minorities’ increased affiliations with their co-ethnics relate to their perceived prejudices or discrimination in the destination country, (e.g. Branscombe et al., 1999; Verkuyten and Yildiz, 2007). Social ties with co-ethnics in the destination country are considered, therefore, a crucial part of minorities’ lives, particularly when they face discrimination and exclusion.

Additionally, the growing body of transnationalism research highlights the importance of transnational co-ethnic social ties of minorities with their friends and families in origin countries (Basch et al., 1994; Faist, 2006; Levitt et al., 2006; Bilgili 2014a, 2014b) as well as the importance of these ties for minorities’ well-being (for a recent review: Horn and Fokkema, 2020). In today’s world, advanced technologies in communication, (e.g. video calling) and transportation enable immigrants to have closer ties and frequent connections with their social networks wherever they are located (Levitt et al., 2006). Therefore, it is important to consider also transnational co-ethnic social ties of minorities, particularly when they face adversity, (e.g. discrimination) in the destination country since these ties can provide them with social support.

Research on coping mechanisms is yet to simultaneously consider social ties that are both locally and transnationally located. By addressing this gap in the literature, we aim to provide a more elaborated and nuanced analysis of minorities’ co-ethnic social ties as coping mechanisms. Furthermore, even though the limited literature on transnational ties showed its importance as a mitigating factor against adversity (see Murphy and Mahalingam, 2004; Snel et al., 2016), less is known about the experiences of the second generation (Åkesson, 2011). This is an important gap as the second generation often has the social skills (e.g., language) and the connections with the origin country of their parents (e.g., via relatives), making transnational ties highly relevant also for them (Levitt, 2009). Furthermore, prior research shows that the second generation is often more aware of discrimination against their ethnic group than the first generation, for instance, due to their more frequent contact with the natives (Heath, 2014). These make it critical to understand how perceived discrimination relates to transnational ties of the second generation. Against this backdrop, we study the relationship between perceived discrimination, (transnational) co-ethnic social ties, and life satisfaction in the Netherlands. By comparing the relevance of these social ties for first- and second-generation minorities in the face of perceived adversity in the destination country, this study extends the literature on transnationalism among the second generation for whom the ontological (in)security of “being from here and there” poses unique challenges as well as opportunities.

Using the Netherlands Longitudinal Life Course Study (NELLS), this article focuses on first- and second-generation Turkish and Moroccan minorities in the Netherlands. It is interesting to study the experiences of these groups as they constitute the two largest non-western groups in the Netherlands (CBS, 2019). Both groups initially migrated to the Netherlands as labour immigrants in the 1960 and 1970s, followed by family migration (Van Londen et al., 2007). As two groups with predominantly Muslim backgrounds, not only their ethnic but also their religious identity plays a role in their perceptions of high levels of discrimination in the Netherlands (Andriessen et al., 2020). Recent data also showed that these perceptions of discrimination are, to a large extent, also part of the lives of the second generation (CBS, 2016; ECRI, 2019; Andriessen et al., 2020).

### Perceived Discrimination, Co-Ethnic Social Ties and Life Satisfaction

Minorities utilize their co-ethnic social ties to receive social support—support that provides them a sense of stability, predictability, and recognition of their self-worth (Cohen and Wills, 1985; Tegegne and Glanville, 2018). When faced with discrimination in the destination country, minorities may feel excluded from the majority group and turn more towards their ethnic group for such support. Building on the well-known Social Identity Theory (SIT) (Tajfel and Turner, 1986), the Rejection-Identification Model (RIM) argues that feelings of threat towards one’s ingroup, (e.g. discrimination) can strengthen individuals’ identification with their ingroup and, relatedly, increase their social relationships with their co-ethnics (Branscombe et al., 1999; Ashmore et al., 2004, pg. 92)^1^. This increased social embeddedness with their co-ethnic can help minorities improve their well-being (Branscombe et al., 1999).

Following this argument, we argue that perceiving discrimination based on ethnicity encourages minorities to build social ties with their co-ethnics to seek support. In this regard, perceived discrimination and co-ethnic ties are not independent, but related to each other. Prior research on coping mechanisms has often examined how minorities utilize their existing co-ethnic ties to help them deal with the adverse consequences of discrimination on their well-being (Sellers and Shelton, 2003; Jasinskaja-Lahti et al., 2006). Adding to this rich strand of research, we aim to study how feeling discriminated against could motivate minorities to turn to their co-ethnics for social connections. This could not only mean strengthening existing co-ethnic social ties but also building new co-ethnic ties to seek support.

The social capital perspective provides a further explanation for the function of social ties as providing social support, particularly when individuals face adverse conditions in society. Putnam (2000) refers to bonding social capital to define social engagement between people belonging to the same social group. This type of capital can provide emotional support and protect self-rated health (Beaudoin, 2009; Tegegne and Glanville, 2018). Following this, Turkish and Moroccan minorities could invest more intensely in their bonding social capital with their co-ethnics to improve their subjective well-being when faced with discrimination or hostility in the destination country.

#### Transnational Co-Ethnic Social Ties

In addition to the theoretical mechanisms provided above, we specifically discuss the relationship between perceived discrimination and transnational co-ethnic social ties with the reactive transnationalism argument. Namely, Itzigsohn and Saucedo (2002) argue that negative experiences, such as discrimination, can increase immigrants’ transnational activities as a reaction. This type of transnationalism can function as an escape from the rejection felt in the destination country and can be compensatory for the social exclusion (Castañeda et al., 2014). Moreover, as research on new communication technologies (e.g. via the Internet) has demonstrated, the modern pattern of technologies does not just substitute face-to-face communication but creates new resources and constructs a new kind of connected presence (Licoppe and Smoreda, 2005). Therefore, transnational social ties with origin country can function as a source of social support that minorities invest in when they feel hostility in the destination country.

As Snel et al. (2016) indicate there is only a handful of studies focusing on reactive transnationalism compared to studies on other consequences of perceived discrimination, (e.g. increased affiliations with one’s co-ethnic group (Branscombe et al., 1999)). For example, in their study conducted in the Dutch context among middle-class immigrants living in Rotterdam, Snel et al. (2016) found that perceiving discrimination based on their ethnicity associates positively with immigrants’ having more transnational social ties. Some qualitative studies conducted among different minority groups in the United Kingdom context also provided similar results regarding minorities’ increased transnational activities when faced with discrimination (Redclift and Rajina, 2019; Pourmedhi, 2020). Therefore, we hypothesize that *Turkish and Moroccan minorities*’ *perceptions of discrimination in the Netherlands associate positively with their transnational co-ethnic social ties* (H1).

Research on transnationalism has been conducted mostly among first-generation minorities (Castañeda et al., 2014; Bilgili, 2014a, 2014b). However, reactive transnationalism could also be relevant for the second generation, especially considering the relatively high levels of discrimination they perceive (Heath, 2014; Andriessen et al., 2020). As Levitt (2009) explains, when second-generation minorities grow up in households influenced by their families’ origin countries, they also socialize into the norms and values of this culture and form social ties across borders. Many second-generation minorities, therefore, have the social skills, (e.g. language skills) and resources, (e.g. via relatives) to form transnational social ties (Levitt, 2009).

Despite their relevance for the lives of the second generation, the extent to which the second generation engages with transnational ties could be lower than the first generation due to two reasons. Firstly, having spent a larger part of their lives in the origin country, the first generation may have stronger interpersonal ties with their friends and relatives living there. In contrast, the second generation may develop these connections indirectly often via their parents or during short visits to the origin country which may lower the intensity and closeness of these ties. Secondly, the skills and resources of the second generation to build transnational ties may be weaker than those of the first generation. For instance, even though second-generation minorities may have some knowledge of the origin-country language, often their proficiency remains lower than the first generation. Accordingly, while transnational ties are still part of the lives of the second generation, both quantitative and qualitative studies have demonstrated that the extent and frequency of transnational social ties may be lower for the second compared to the first generation (Viruell-Fuentes, 2006; Wessendorf, 2016). Furthermore, as Beauchemin et al. (2011) show perceiving discrimination was one of the main factors encouraging second-generation minorities from France to form transnational social ties. We hypothesize that perceived discrimination associates positively with transnational co-ethnic social ties also for the second-generation Turkish and Moroccan minorities although the strength of this association for the second generation is weaker than for the first-generation (H2).

**FIGURE 1:**
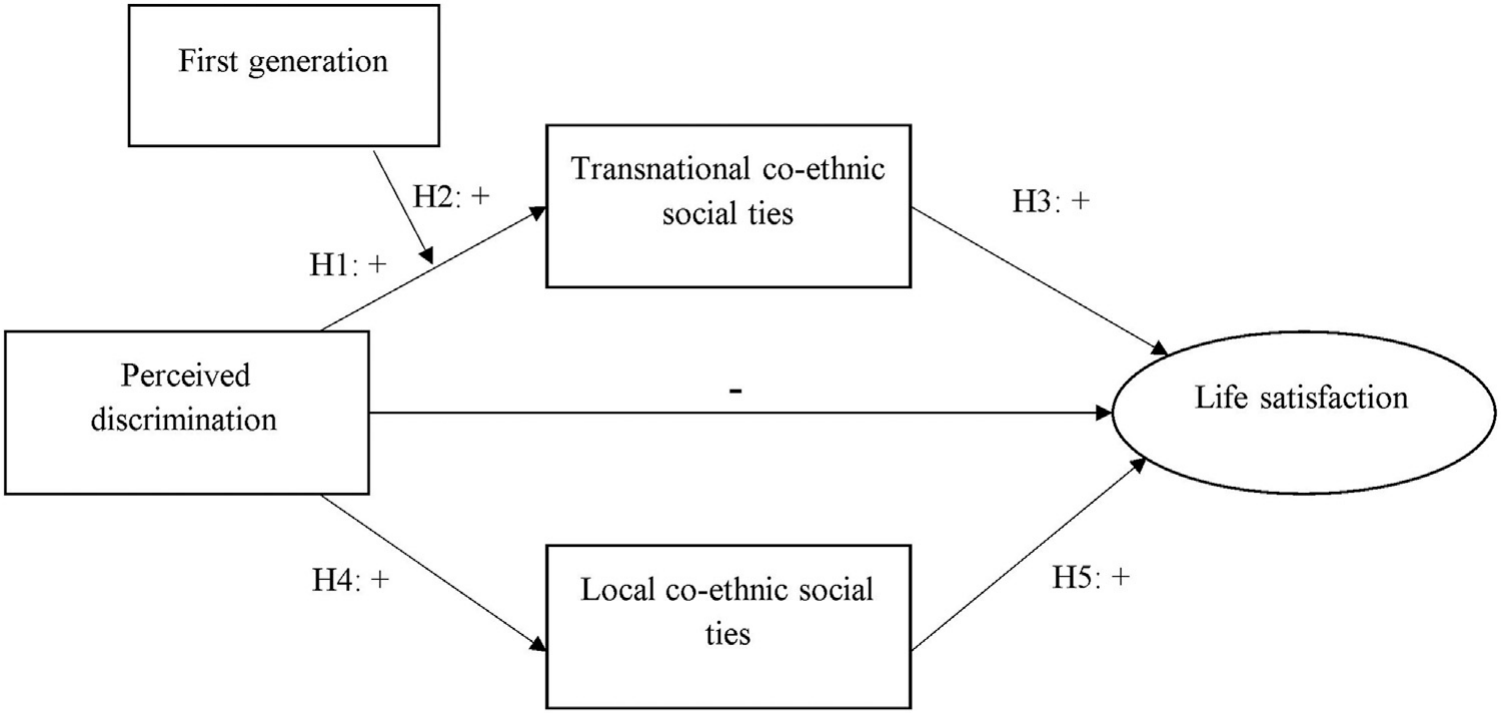
Theoretical model. Control variables: Age, gender, employment, Dutch language skills, education, financial difficulties, share of co-ethnic neighbours.

Prior research suggests that transnational social ties with family and friends may have both positive and negative impacts on the subjective well-being and mental health of ethnic minorities (Torres et al., 2016; Horn and Fokkema, 2020). On the one hand, transnational social ties can be a stress factor as they may induce feelings of social obligation and emotions of separation. On the other hand, since these are ties transcending national boundaries, they can facilitate minorities’ belonging within a broader family which makes minorities feel understood, valued and cared for by others,—which could, in turn, increase their well-being (Torres, 2013). Furthermore, through these types of social ties, minorities can compare their quality of life in the destination country with their family and friends abroad. Particularly in the context of migration from economically less developed to more developed countries, these comparisons may be positive giving a better sense of well-being to minorities (Alcántara et al., 2014; Torres et al., 2016). Nieswand (2011) also argues that they may develop an awareness of their privileged socio-economic situation compared to those left-behind. In line with these arguments, research conducted among Caribbean immigrants has shown that transnational social ties with origin countries are positively related to immigrants’ life satisfaction (Murphy and Mahalingam, 2004). While recognizing potential stress factors related to transnational social ties, following these results, we hypothesize that *increased transnational co-ethnic social ties of Turkish and Moroccan minorities in their origin countries associate positively with their life satisfaction* (H3). The theoretical model can be seen in Figure 1.

#### Local Co-Ethnic Social Ties

As local sources that are geographically proximate to ethnic minorities, local co-ethnic social ties in the destination country are vital elements in providing social support against loneliness and isolation and mainly to give first-hand support (Ryan, 2007; Ryan et al., 2008). Therefore, when ethnic minorities perceive themselves as being discriminated against, they can immediately turn to their co-ethnics in the destination country to receive support. Building on this literature, we hypothesize that *perceived discrimination of Turkish and Moroccan minorities in the Netherlands associate positively with their local co-ethnic social ties in the destination country* (H4). As both first- and second-generation minorities live in the destination country, the extent of their skills and opportunities to build local co-ethnic social ties are more similar than those to build transnational ones. We, therefore, do not expect generation differences regarding local co-ethnic social ties.

In the lives of ethnic minorities, having social ties with their co-ethnics who have similar experiences may have various supportive roles. The social support provided by people who have similar experiences could be useful since it can lead minorities to feel “empathetic understanding” which is important in coping with negative experiences (Thoits, 1986). This type of understanding could give them the feeling that their experiences and emotions are understood by people who perceive similar difficulties. Furthermore, local co-ethnic social ties could give minorities the chance to observe their co-ethnics reactions when they face a similar disadvantaged situation in the destination country. Seeing people who have coped with similar difficulties can help individuals develop active coping behaviours, reduce stress, and manage their emotional reactions (Thoits, 2011). Consequently, we hypothesize that *increased local co-ethnic social ties of Turkish and Moroccan minorities in the Netherlands relate positively to their life satisfaction* (H5).

### Data and Methods

#### Data and Participants

This study used data from the first wave of the Netherlands Longitudinal Life Course Study (NELLS) (De Graaf et al., 2010a). As the questions on minorities’ transnational co-ethnic social ties were only in this wave, we used this wave of the data. NELLS was designed as a panel survey for sociological themes, mainly related to social cohesion, norms, values, and inequality (De Graaf et al., 2010b). This survey targeted people aged 15–45 and applied a two-stage stratified sampling procedure. Firstly, 35 municipalities (including 256 neighbourhoods) were randomly selected by region and urbanization. Later on, respondents were randomly chosen based on their age and ethnic origins from these municipalities. There was an initial sample of 5,312. Respondents from Turkish and Moroccan minority groups were oversampled. The data were collected using both face-to-face and self-completion methods. The data included a small sample of missing values (7.7%) due to the self-completion part. We excluded these missing values as some of our questions came from this part of the survey. Furthermore, due to the focus of our article, we only chose respondents from Turkish and Moroccan minority groups, which resulted in a final sample of 2012 respondents. This sample included 646 first-generation, 358 second-generation Turkish minorities, and 638 first-generation, 370 second-generation Moroccan minorities.

### Measures

In the present study, the *dependent variable* was life satisfaction. This measure was constructed as a reflective latent variable composed of four statements. These statements were “my life is for most parts ideal,” “my living conditions are excellent,” “overall, I am content with my life” and “the most important things I expect from life I achieved so far." They had five answer categories ranging from “strongly agree” to “strongly disagree." All the items were reverse coded before constructing the latent variable so that a higher score indicated higher life satisfaction. The resulting latent variable was found reliable for the whole sample and for the sub-groups according to ethnic groups and generations.^2^

The *independent variable* of the study was perceived discrimination. To measure this, we constructed a formative latent variable by taking the average of the questions asking respondents if they ever felt discriminated on the basis of their ethnic origin at work, school, in class, on the street, in public transport, associations, clubs, sports and at nightlife separately. These questions had the answer categories of “never,” “yes, only once,” “yes, often.” A higher score indicated higher levels of perceived discrimination.

The *first mediator* was transnational co-ethnic social ties with origin country. It was also constructed as a formative latent variables with the questions of “How often have you visited the country of origin of your parents in the last 12 months?” and “How often have you had contact via phone or email with family or friends in that country in the last 12 months?” These questions had the answer categories of “yes, several times,” “yes, once in a while” and “no.” They were reverse coded so that a higher score on this variable indicated more frequent transnational co-ethnic social ties with the origin country.

The *second mediator* was local co-ethnic social ties in the destination country. It was taken as a formative latent variable composed of the questions on how often respondents have contacts with their co-ethnics in the neighbourhood, at work/school and in leisure clubs in the Netherlands. The questions had answer categories ranging from 1 (“every day”) to 7 (“never”) which were reverse-coded so that a higher score showed more frequent contact with co-ethnics in the Netherlands.

Ethnicity and generation were used as *grouping variables* in multi-group analyses, comparing first and second generations from Turkish and Moroccan minority groups. Following the definition of ethnicity and generation of Statistics the Netherlands (CBS 2016), ethnicity was classified based on the countries of birth of the respondents and both their parents. Accordingly, if the person and one or two parents were born outside the Netherlands, the respondent was classified as “first generation” whereas if the person was born in the Netherlands while one or two parents were not, that person was taken as “second generation” (De Graaf et al., 2010b).

Literature shows that perceptions of discrimination and involvement in transnational practices show differences between gender groups (Itzigsohn and Saucedo, 2002; Alanya et al., 2017). Furthermore, financial situation relates to the capacity to engage in transnational activities (Snel et al., 2016). Additionally, ethnic minorities’ social ties could be related to their Dutch language skills (Martinovic et al., 2008) and the share of co-ethnics in the neighbourhood (Huijts et al., 2014). Following this prior research, we controlled for the effects of age, gender (1 = “Female”, 0 = “Male”), being employed (1 = “Yes”, 0 = “No”), Dutch language proficiency (from 1 = “None*”* to 5 = “Very good*”*), educational attainment, financial difficulties, and share of co-ethnics in the neighbourhood (in percentages). While constructing the variable of educational attainment variable, for those who are still in education, we took their current level of education, whereas, for those who have left education, we took their highest level of education attainment (1 = “low education”–6 = “tertiary education”). The variable of financial difficulties was a scale variable (0–1) constructed by taking the average of five questions such as “in the last 3 months, whether you have had difficulties making ends meet.” Higher scores on this measure indicated having more financial difficulties.

### Analyses

All the missing values -around 5 %- were handled using full information maximum likelihood (FIML) method in Mplus. We constructed most variables as formative latent variables since respondents who scored high on different items within this latent variable should also score high on this latent variable even if these items were not correlated with each other. One of the aims of this study was to compare the path from perceived discrimination to transnational co-ethnic social ties for first- and second-generation Turkish and Moroccan minorities. We, therefore, conducted a moderated mediation analysis. We did so by conducting a multi-group structural equation modelling for the groups of first- and second-generation Turkish and Moroccan minorities.

Since the present study contained one reflective latent variable -life satisfaction-, measurement invariance across these four groups was performed. Later on, structural invariance tests were performed in order to obtain a final structural model that enables testing mediation paths for different groups in the analysis. As our model was complicated enough with reflective and formative latent variables, the complex survey method was chosen in order to take the nested structure of the data into consideration (see Asparouhov and Muthen, 2006).

## Results

### Measurement Model

In the present analysis, one reflective latent variable was constructed to measure “life satisfaction." Measurement invariance tests showed that this latent variable had a comparable fit across the ethnic groups and generations in the analysis (see Tables 1, 2). For the formative latent variables in the analysis, equality of weights of indicators for each of these variables was assumed across the groups.

**TABLE 1:** Measurement invariance test for CFA (1) for “life satisfaction” with four items across ethnic minority groups and generations (*N* = 2012).

Invariance	SB-Chi2 (df)	RMSEA	CFI	TLI	SRMR	SB- Chi2 (df), (p)
Configural	23.443 (8) **	0.062	0.994	0.981	0.012	–
Metric	29.667 (17) *	0.039	0.995	0.993	0.040	7.0826 (9), *p* = 0.629
Scalar	44.951 (26) *	0.038	0.992	0.993	0.046	20.9693 (18), *p* = 0.2810

Note: SB stands for Satorra-Bentler Chi-square difference test. *p < 0.05; **p < 0.01 (two-tailed).

**TABLE 2:** Unstandardized factor loadings of the latent factor of “life satisfaction.”

Items	Factor loadings
1. My life is for most parts ideal	1
2. My living conditions are excellent	1.053***
3. Overall, I am content with my life	0.914***
4. The most important things I expect from life I achieved so far	0.963***

***p < 0.001 (two-tailed).

### Structural Model

We performed structural invariance tests to obtain a well-fitting structural model allowing us to test our hypotheses across the ethnic and generation groups in our study. These tests showed that a partially constrained model had a reasonably good fit comparable to that of an unconstrained model (see Table 3). We reached this partially constrained model by allowing the path from “transnational co-ethnic social ties” to “perceived discrimination” to vary across the generation groups—but not across ethnic groups -, in line with our hypothesis on generational differences. This model, therefore, allowed us to keep this specific mediation path comparable across the ethnic groups but not across the generations. As a result, in the partially constrained model, the mediation paths were statistically comparable between Turkish and Moroccan minority groups and between the first and second generations, except for the generational difference for the path from “transnational co-ethnic social ties” to “perceived discrimination.” As the partially constrained model is more parsimonious, this model was taken as the final model to test our hypotheses.

**TABLE 3:** Structural invariance test between 1^st^ generation Turkish minority (*n* = 624), 2^nd^ generation Turkish minority (*n* = 350), 1^st^ generation Moroccan minority (*n* = 620) and 2^nd^ generation Moroccan minority (*n* = 351) (*N* = 2012).

	SB-Chi2 (df)	RMSEA	CFI	TLI	SRMR	SB Chi2 difference, (df
Unconstrained	222.828 (146) **	0.032	0.982	0.969	0.023	–
Constrained	249.660 (161) ***	0.033	0.979	0.968	0.027	28.8608 (15), *p* = 0.030
Partially constrained	240.731 (160) **	0.032	0.981	0.971	0.026	17.9429 (14), *p* = 0.209

Notes: In partially constrained model, the path from “perceived discrimination” to “origin-country co-ethnic social ties” were let to vary across generations, but constrained to be the same across ethnic groups within generations. **p < 0.05, ***p < 0.001 (two-tailed).

### Descriptive Results

Descriptive results are presented in Table 4. The second generation reported having higher life satisfaction than the first generation, for both Turkish and Moroccan ethnic groups (F (3, 2008) = 8.260, *p* < 0.001). Nevertheless, for each group, the levels of life satisfaction were significantly above the midpoint of scale (see Supplementary Table S1). This meant that, on average, the respondents defined their satisfaction with life positively, although not indicating that they were completely satisfied. For perceived discrimination, second-generation Moroccan minorities had the highest average, meaning that they perceived it the most (F (3, 1961) = 6.171, *p* < 0.001). However, the means of all the groups for this variable were significantly below the midpoint of the scale, indicating that most respondents did not perceive discrimination to a large extent (see Supplementary Table S1; Supplementary Appendix). Accordingly, 36 percent of the respondents from all groups said that they have never perceived discrimination.

**TABLE 4:** Descriptive statistics of all variables in the analyses [1^st^ generation Turkish minority (*n* = 646), 2^nd^ generation Turkish minority (*n* = 358), 1^st^ generation Moroccan minority (*n* = 638), 2^nd^ generation Moroccan minority (*n* = 370)].

	Range	Mean (SD)			
		1st gen Turkish minority	2nd gen Turkish minority	1st gen moroccan minority	2nd gen moroccan minority
Dependent variable					
Life satisfaction	1–5	3.55 (0.76)^a^	3.67 (0.69)^b^	3.62 (0.80)^a^	3.78 (0.68)^b^
Independent variable					
Perceived discrimination	1–3	1.34 (0.41)^a^	1.30 (0.38)^a^	1.32 (0.39)^a^	1.42 (0.44)^b^
Mediators					
Transnational ties	1–3	2.24 (0.56)^a^	2.22 (0.62)^a^	2.18 (0.56)^a^	2.15 (0.63)^a^
Local ties	1–7	4.53 (1.60)^a^	4.82 (1.59)^b^	4.26 (1.56)^c^	4.96 (1.67)^b^
Control variables					
Age	14–49	35.57 (7.51)	24.70 (7.48)	34.40 (6.94)	22.98 (6.58)
Female	0/1	0.49	0.55	0.54	0.56
Dutch Language proficiency	1–5	3.80 (1.05)	4.62 (0.64)	4.04 (1.01)	4.84 (0.39)
Education	1–6	2.79 (1.68)	3.27 (1.36)	2.74 (1.63)	3.43 (1.29)
Financial difficulties	0–1	0.23 (0.29)	0.18 (0.26)	0.23 (0.28)	0.13 (0.22)
Employment	0/1	0.64	0.59	0.60	0.60
Share of co-ethnic neighbors	1–31	7.28 (6.91)	7.49 (6.47)	9.47 (7.24)	9.03 (7.32)

Notes: For mean values with the same superscript in each row shows that mean values for those groups are not statistically significantly different from each other. In order to conduct the post-hoc test of one-way ANOVA, the mediators–transnational and local ties- and dependent variable–life satisfaction- (which is originally a reflexive latent variable) were taken as an average variable and the mean values were reported from SPSS based on this construction for these variables. Standard deviations are not reported for dichotomous variables.

Regarding transnational co-ethnic social ties, there was no significant difference between the groups (F (3, 1964) = 2.397, *p* = 0.07). For both ethnic groups and generations, the mean values were slightly higher than the midpoint of the scale. This meant that, on average, respondents had relatively frequent social ties with their origin countries. For local co-ethnic social ties, the second generation from both ethnic groups had more frequent contacts with their co-ethnics in the Netherlands compared to the first generation (F (3, 2002) = 18.186, *p* < 0.001). Among the first generation, the Turkish group had more frequent local ties than the Moroccan group. For all the groups, the mean values were significantly and slightly above the midpoint of the scale. This indicated that most respondents had contact with their co-ethnics more than once a month.

### Main Results

**FIGURE 2:**
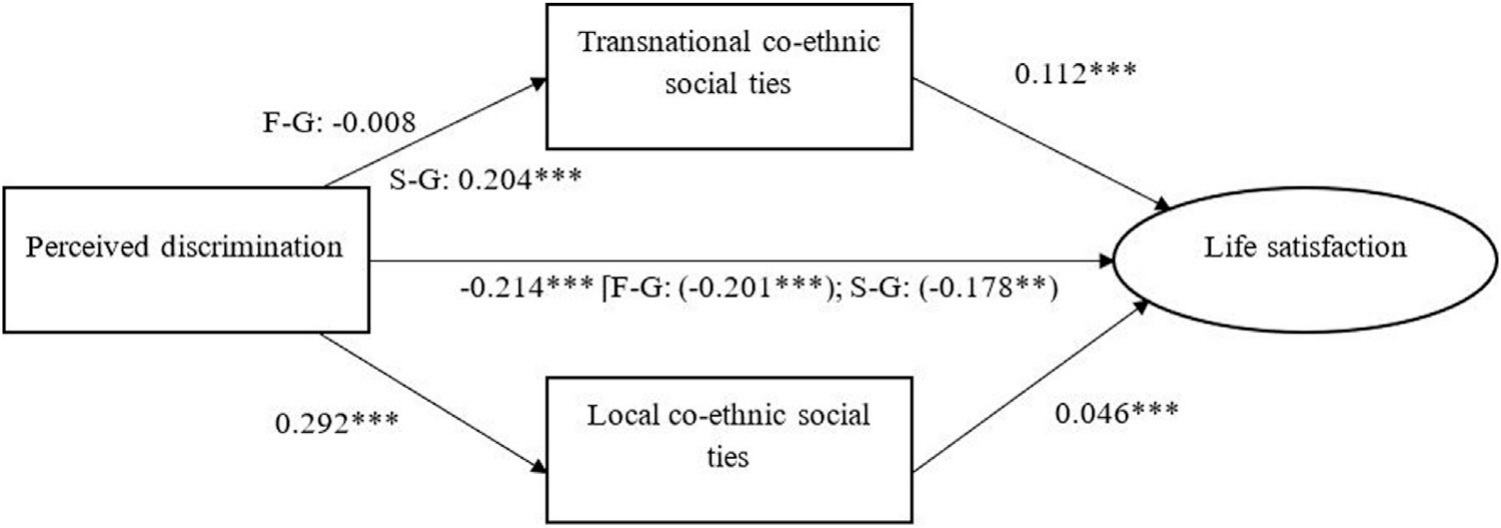
Path diagram of directing and meditating first and second generation Turkish Morrocon Minorities. Note: F-G and S-G are stands for first and second generation respectievly. Total effect are reported for two groups in the parenthesis.Reflective latent variables are represent in the circle **p < 0.01,***p < 0.001(two tailed).

The results of the partially constrained model are presented in Figure 2 (also see Supplementary Tables S2, S3 in Supplementary Appendix). These results showed that perceiving ethnic-based discrimination had a negatively direct association with minorities’ life satisfaction. In line with the first hypothesis, for second-generation Turkish and Moroccan minorities, perceived discrimination is associated positively with their transnational co-ethnic social ties. However, this was not the case for the first generation as the path from transnational ties to perceived discrimination was not statistically significant for this group. The first hypothesis, therefore, was partially supported. With the second hypothesis, we expected the association between perceived discrimination and transnational ties to be stronger for the first generation than the second generation. Wald tests showed that the coefficients for this path indeed differed from each other for the two generations (Wald Chi2 (1) = 11.757, *p* < 0.0001). The fact that this path was not statistically significant for the first generation, while it was for the second generation, could mean that there was a stronger association between perceived discrimination and transnational social ties for the second than for the first generation. Therefore, we rejected our second hypothesis.

In line with our third hypothesis, increased transnational co-ethnic social ties of Turkish and Moroccan minorities associated positively with their life satisfaction for both generations. Despite the statistically significant path from transnational social ties to life satisfaction, the indirect effect of perceived discrimination on life satisfaction via these ties was not statistically significant for the first generation (b = −0.001, *p* = 0.831) while it was for the second generation (b = 0.023, *p* = 0.005).

Regarding the role of local co-ethnic social ties, we found support for our fourth hypothesis predicting that perceived discrimination associates positively with local co-ethnic social ties in the destination country. The analysis yielded a positive association between perceiving discrimination and having local co-ethnic social ties. With the fifth hypothesis, we further expected increased local co-ethnic social ties to associate positively with their life satisfaction. This hypothesis was also supported by the analysis. As a result, the indirect effect of perceived discrimination on life satisfaction was statistically significant (b = 0.013 *p* = 0.002). This meant that perceived discrimination was associated with minorities' increased social ties with their co-ethnics in the Netherlands. And that these ties, in turn, were beneficial for their life satisfaction.

## Discussion

Ethnic origin is one of the main determinants of perceived discrimination in the Netherlands (Andriessen et al., 2020). Research has demonstrated that perceptions of discrimination negatively relate to minorities’ life satisfaction (Verkuyten, 2008). Building on this research, in this article, we aimed to analyse the extent to which minorities utilize their co-ethnic social ties in different geographical locations to mitigate the negative effects of perceived discrimination on their life satisfaction. We focused on Turkish and Moroccan minorities in the Netherlands. As the largest non-western groups in the Netherlands, both groups have disadvantaged socio-economic status in the Netherlands and perceive relatively high levels of discrimination which also applies to the second generation to a large extent (CBS, 2016; ECRI, 2019; Andriessen et al., 2020).

By focusing on minorities’ co-ethnic social ties located in different countries simultaneously, we presented a more elaborate investigation of how these social ties suppress the negative effects of perceived discrimination on life satisfaction. Moreover, we compared first- and second-generation minorities based on how they utilize their transnational co-ethnic social ties in the face of adversity in the destination country. Finally, this study brought together varying strands of literature on transnationalism, perceived discrimination, and psychological well-being to understand better the experiences of Turkish and Moroccan minorities in the Netherlands.

In line with previous literature, we found that perceived discrimination was negatively related to the life satisfaction of Turkish and Moroccan minorities in the Netherlands (e.g. Schmitt et al., 2003)—though we should note that our respondents perceived, on average, relatively low discrimination. Furthermore, we showed that both generations had rather frequent transnational social ties with their origin countries. Perceived discrimination, however, was positively related to having transnational ties only for the second generation. As Levitt (2009) argues, second-generation minorities often grow up in households with ideological, material, or affective connections to the origin country. They, therefore, have the social skills to engage with the origin country. In line with reactive transnationalism (Itzigsohn and Saucedo, 2002), our findings showed that negative contexts, such as discrimination, in the destination country could motivate the second generation to use these social skills to form more transnational social ties, (e.g. Portes and Rumbaut, 2001; Fokkema et al., 2012). This way, they could feel more social support in their lives. The absence of the relation between perceiving discrimination and forming transnational ties for the first generation could be due to the first generation minorities having ties with their origin countries mainly because they lived there (Beauchemin et al., 2011). Hence, for the first generation, having these transnational ties might not be driven primarily by their (negative) experiences in the destination country.

For both generations, having more transnational co-ethnic social ties was, in turn, related to higher levels of life satisfaction. This result supports the benefits of having these ties for psychological well-being, (e.g. Torres, 2013). Hence, our findings indicate that while having social ties with their origin countries is positively associated with the life satisfaction of both generations, it functions as a coping mechanism only for second-generation Turkish and Moroccan minorities in the Netherlands.

Regarding minorities’ local co-ethnic social ties in the destination country, both Turkish and Moroccan minorities had rather frequent contact with their co-ethnics living in the Netherlands. The present study demonstrates that these co-ethnic social ties could help both generations to deal with the negative influences of perceived discrimination on their psychological well-being. This finding emphasized the importance of geographically close co-ethnic ties provide social support, (e.g. Ryan, 2007). Furthermore, it showed that those who have similar negative experiences could understand the difficulties associated with those experiences and help each other manage the negative consequences, (e.g. Thoits, 2011).

## Conclusion

Minorities perceive discrimination based on their ethnicity in different areas of their lives (ECRI, 2019; Andriessen et al., 2020). In this article, we showed that these perceptions of discrimination have negative implications for ethnic minorities’ life satisfaction. The fact that life satisfaction is an important dimension of psychological well-being makes it even more pressing to tackle the issue of continuing ethnic discrimination in many destination countries. In this regard, not only actual experiences of discrimination but also perceptions or feelings of discrimination as a reflection of these negative experiences are crucial to targeting to achieve better well-being outcomes for ethnic minorities.

As a way of tackling the negative influences of perceived discrimination on well-being, we showed the importance of minorities’ transnational and local co-ethnic social ties as two channels through which the negative effects of perceived discrimination on life satisfaction are decreased. It is particularly interesting to see the role of transnational social ties as a coping mechanism, especially with regards to the second generation. Our results demonstrated that minorities utilize their transnational social ties as well as their local co-ethnic social ties while being settled in the destination country, when they face adversity in this country. This means that in the investigation of minorities’ experiences in the Netherlands and their overall well-being, it is necessary to take into account their co-ethnic social ties and networks as a whole, independent of where they are located. This provides a more holistic understanding of minorities’ lives which can eventually help determine the best ways to support them and increase their well-being in the face of continuing ethnic discrimination.

In short, this research aimed at contributing to the literature on coping mechanisms against perceived discrimination among ethnic minorities. Extending the literature on previously studied coping mechanisms such as problem-solving or positive reappraisal (Tandon et al., 2013), we focused on seeking social support among both locally and transnationally located co-ethnics. Despite its important findings, our research had some limitations. Firstly, our focus was only on Turkish and Moroccan minority groups in the Netherlands. These groups are large and established ethnic minority groups in the Netherlands. Future research could test the role (transnational) co-ethnic social ties among smaller and newer ethnic minority groups in other contexts. Particularly, the role of local co-ethnic social ties may be different for these groups due to the lack of a large ethnic community in the destination country. Secondly, our measures of transnational and local social ties were not comparable to each other—preventing us from comparing the extent to which minorities utilize them. And, lastly, our study had a restricted measure of transnational ties. Future research could use more comprehensive and comparable measures of locally and transnationally located co-ethnic social ties (e.g., how important people consider these ties to be). Using these measures would also ultimately contribute to methodological and operational advancements within well-being and transnational migration studies.

## Data Availability

The dataset analysed during the current study is available in DANS repository, https://easy.dans.knaw.nl/ui/datasets/id/easy-dataset:34387.

## Footnotes

1 The present article refers to the literature on strong group identity as a coping mechanism against negative effects of perceiving discrimination. It is, however, important to acknowledge that strong ethnic identity can also function as a risk factor and increase ethnic minorities’ perceptions of discrimination, (e.g. Sellers and Shelton, 2003).

2 Composite reliability for the full sample: rho = 0.852; for first-generation Turkish minorities: rho = 0.857; for second-generation Turkish minorities: rho = 0.834; for first-generation Moroccan minorities: rho = 0.875; for second-generation Moroccan minorities: rho = 0.802.
