# Corrigendum: Transnational and Local Co-ethnic Social Ties as Coping Mechanisms Against Perceived Discrimination - A Study on the Life Satisfaction of Turkish and Moroccan Minorities in the Netherlands

**DOI:** 10.3389/fsoc.2022.856073

**Published:** 2022-02-14

**Authors:** Ece Arat, Özge Bilgili

**Affiliations:** ^1^Department of Sociology, Faculty of Social and Behavioural Sciences, Utrecht University, Utrecht, Netherlands; ^2^European Research Centre on Migration and Ethnic Relations (ERCOMER), Faculty of Social and Behavioural Sciences, Utrecht University, Utrecht, Netherlands

**Keywords:** perceived discrimination, coping mechanisms, transnational co-ethnic social ties, local co-ethnic social ties, the Netherlands, life satisfaction

In the original article, there was a mistake in the legend for “Figure 1” as published. “Not all control variables in the analyses were fully mentioned in the Figure legend.” The correct legend appears below.

**Figure 1:**
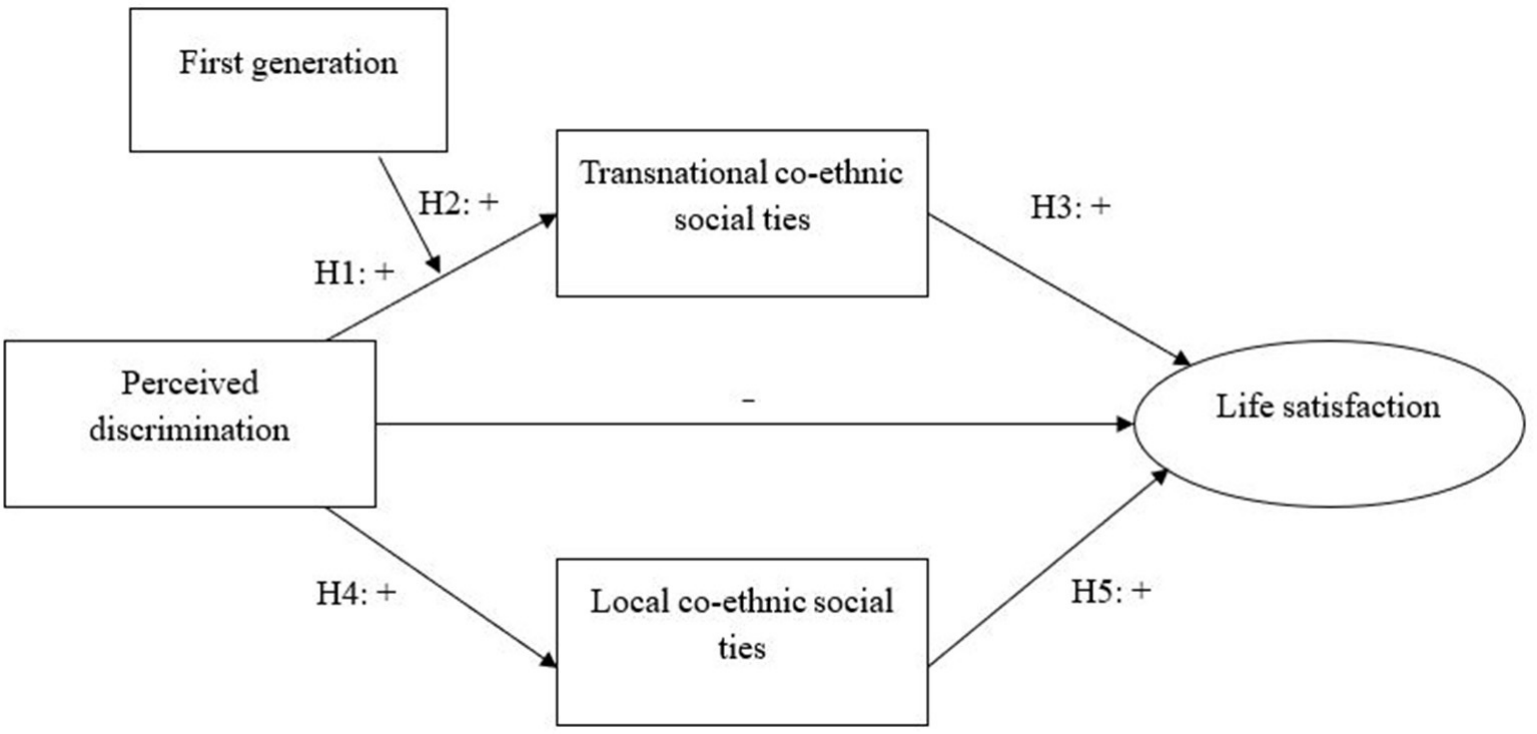
Theoretical model. Control variables: Age, gender, employment, Dutch language skills, education, financial difficulties, share of co-ethnic neighbours.

In the original article, the reference for “Asparouhov and Muthen, 2006” was incorrectly written as “Asparouhov, T., and Muthen, B. (2006). *Comparison of Estimation Methods for Complexsurvey Data Analysis. Mplus Web Notes*.” The correct reference appears below.

In the original article, the reference for “Basch et al., 1994” was incorrectly written as “Basch, L. G., Glick-Schiller, N., and Szanton Blanc, C. (1994). *Nations Unboundtransnational Projects, Postcolonial Predicaments, and Deterritorialized Nation-States*. Langhorne: Gordon & Breach. http://www.statmodel.com/resrchpaps.html.” The correct reference appears below.

The authors apologize for this error and state that this does not change the scientific conclusions of the article in any way. The original article has been updated.

## Publisher's Note

All claims expressed in this article are solely those of the authors and do not necessarily represent those of their affiliated organizations, or those of the publisher, the editors and the reviewers. Any product that may be evaluated in this article, or claim that may be made by its manufacturer, is not guaranteed or endorsed by the publisher.
