# Supplementary material for: Transnational and Local Co-ethnic Social Ties as Coping Mechanisms Against Perceived Discrimination - A Study on the Life Satisfaction of Turkish and Moroccan Minorities in the Netherlands
Source: Front Sociol. 2021 Jun 28;6:671897. doi: 10.3389/fsoc.2021.671897 (PMC8273254; doi:10.3389/fsoc.2021.671897)
Supplement: Supplementary file 1 [file Table1.docx]

**Supplementary Material**

**Table 1.** One- sample T-test for each ethnic and generation groups [1^st^ generation Turkish (n=646), 2^nd^ generation Turkish (n=358), 1^st^ generation Moroccan (n=638), 2^nd^ generation Moroccan minorities (n=370)].

|  |  |  | t (df) |  |
| --- | --- | --- | --- | --- |
|  | 1^st^ gen Turks | 2^nd^ gen Turks | 1^st^ gen Moroccans | 2^nd^ gen Moroccans |
| ***Dependent variable*** |  |  |  |  |
| Life satisfaction | 18.242 (645) *** | 18.499 (357) *** | 19.761 (637) *** | 22.134 (369) *** |
| ***Independent Variable*** |  |  |  |  |
| Perceived discrimination | -39.912 (630) *** | -34.370 (351) *** | -43.072 (628) *** | -25.002 (352) *** |
| ***Mediators*** |  |  |  |  |
| Transnational ties | 10.696 (632) *** | 6.723 (351) *** | 7.952 (628) *** | 4.342 (353) *** |
| Local ties | 8.344 (643) *** | 9.734 (357) *** | 4.265 (635) *** | 11.060 (367) *** |

*** p < .001 (two-tailed).
