# Supplementary material for: Transnational and Local Co-ethnic Social Ties as Coping Mechanisms Against Perceived Discrimination - A Study on the Life Satisfaction of Turkish and Moroccan Minorities in the Netherlands
Source: Front Sociol. 2021 Jun 28;6:671897. doi: 10.3389/fsoc.2021.671897 (PMC8273254; doi:10.3389/fsoc.2021.671897)
Supplement: Supplementary file 2 [file Table2.docx]

**Table 2.** Total, direct, and indirect effects of structural equation model including control variables for first- and second- generation Turkish minorities (N= 2012).

|  |  | First-generation |  |  | Second-generation |  |
| --- | --- | --- | --- | --- | --- | --- |
|  | Transnational social ties (SE) | Host-country co- ethnic social ties (SE) | Life satisfaction (SE) | Transnational social ties (SE) | Host-country co- ethnic ties  (SE) | Life satisfaction (SE) |
| ***Total effects*** |  |  |  |  |  |  |
| Perceived discrimination | - | - | -0.201 (0.051) *** | - | - | -0.178 (0.052) ** |
| ***Direct effects*** |  |  |  |  |  |  |
| Life satisfaction | 0.112 (0.029) *** | 0.046 (0.011) *** | - | 0.112 (0.029) *** | 0.046 (0.011) *** | - |
| Perceived discrimination | - 0.008 (0.035) | 0.292 (0.071) *** | -0.214 (0.050) *** | 0.204 (0.052) *** | 0.292 (0.071) *** | - 0.214 (0.050) *** |
| Age | - 0.002 (0.004) | -0.031 (0.009) ** | -0.005 (0.004) | -0.021 (0.004) *** | - 0.083 (0.009) *** | 0 (0.005) |
| Female | 0.045 (0.050) | -0.205 (0.144) | 0.077 (0.064) | 0.120 (0.061) | - 0.560 (0.136) *** | - 0.055 (0.072) |
| Dutch proficiency | -0.100 (0.021) *** | 0.006 (0.050) | 0.083 (0.027) ** | - 0.091 (0.043) * | - 0.205 (0.126) | 0.085 (0.056) |
| Education | 0.070 (0.014) *** | 0.037 (0.047) | - 0.014 (0.018) | 0.024 (0.028) | - 0.022 (0.062) | 0.023 (0.025) |
| Financial difficulties | - 0.187 (0.083) * | - 0.114 (0.243) | - 0.665 (0.096) *** | - 0.037 (0.110) | - 0.148 (0.311) | - 0.946 (0.141) *** |
| Employment | 0.127 (0.059) * | 0.672 (0.151) *** | 0.125 (0.067) | - 0.047 (0.067) | 0.162 (0.154) | 0.068 (0.082) |
| Share of co-ethnic neighbors | -0.005 (0.003) | 0.011 (0.008) | 0.003 (0.005) | 0.004 (0.005) | 0.032 (0.010) ** | 0.001 (0.005) |
| ***Indirect effects*** |  |  |  |  |  |  |
| Via transnational ties | - | - | -0.001 (0.004) | - | - | 0.023 (0.008) ** |
| Via local ties | - | - | 0.013 (0.004) ** | - | - | 0.013 (0.004) ** |

**p* < .05, ** *p* < .01, *** *p* < .001 (two-tailed).
