# Supplementary material for: Transnational and Local Co-ethnic Social Ties as Coping Mechanisms Against Perceived Discrimination - A Study on the Life Satisfaction of Turkish and Moroccan Minorities in the Netherlands
Source: Front Sociol. 2021 Jun 28;6:671897. doi: 10.3389/fsoc.2021.671897 (PMC8273254; doi:10.3389/fsoc.2021.671897)
Supplement: Supplementary file 3 [file Table3.docx]

|  |  | First-generation |  |  | Second-generation |  |
| --- | --- | --- | --- | --- | --- | --- |
|  | Transnational social ties (SE) | Host-country co- ethnic social ties (SE) | Life satisfaction (SE) | Transnational social ties (SE) | Host-country co- ethnic ties  (SE) | Life satisfaction (SE) |
| ***Total effects*** |  |  |  |  |  |  |
| Perceived discrimination | - | - | -0.201 (0.051) *** | - | - | -0.178 (0.052) ** |
| ***Direct effects*** |  |  |  |  |  |  |
| Life satisfaction | 0.112 (0.029) *** | 0.046 (0.011) *** | - | 0.112 (0.029) *** | 0.046 (0.011) *** | - |
| Perceived discrimination | - 0.008 (0.035) | 0.292 (0.071) *** | -0.214 (0.050) *** | 0.204 (0.052) *** | 0.292 (0.071) *** | - 0.214 (0.050) *** |
| Age | - 0.001 (0.004) | -0.028 (0.009) ** | -0.014 (0.004) *** | -0.027 (0.006) *** | - 0.122 (0.016) *** | 0 (0.006) |
| Female | -0.026 (0.050) | -0.193 (0.148) | 0.092 (0.067) | 0.114 (0.073) | - 0.736 (0.159) *** | 0.013 (0.073) |
| Dutch proficiency | -0.062 (0.026) * | 0.026 (0.058) | -0.010 (0.031) | - 0.097 (0.078) | - 0.029 (0.224) | 0.077 (0.082) |
| Education | 0.038 (0.016) * | 0.004 (0.047) | - 0.020 (0.021) | 0.005 (0.027) | 0.004 (0.063) | 0.025 (0.035) |
| Financial difficulties | - 0.237 (0.078) ** | - 0.495 (0.223) * | - 0.770 (0.113) *** | 0.080 (0.148) | 0.254 (0.339) | - 1.117 (0.174) *** |
| Employment | -0.068 (0.050) | 0.567 (0.136) *** | 0.059 (0.073) | 0.017 (0.075) | 0.069 (0.165) | -0.060 (0.081) |
| Share of co-ethnic neighbors | 0.006 (0.003) | 0.016 (0.009) | -0.008 (0.003) * | 0.009 (0.005) | 0.028 (0.012) * | -0.008 (0.005) * |
| ***Indirect effects*** |  |  |  |  |  |  |
| Via transnational ties | - | - | -0.001 (0.004) | - | - | 0.023 (0.008) ** |
| Via local ties | - | - | 0.013 (0.004) ** | - | - | 0.013 (0.004) ** |

**Table 3.** Total, direct, and indirect effects of structural equation model including control variables for first- and second- generation Moroccan minorities (N= 2012).

**p* < .05, ** *p* < .01, *** *p* < .001 (two-tailed).
